# Psychological Determinants of Collective Action for Climate Justice: Insights From Semi-Structured Interviews and Content Analysis

**DOI:** 10.3389/fpsyg.2021.695365

**Published:** 2021-07-28

**Authors:** Hannah Bührle, Joachim Kimmerle

**Affiliations:** ^1^Department of Psychology, University of Tuebingen, Tuebingen, Germany; ^2^Leibniz-Institut fuer Wissensmedien/Knowledge Media Research Center, Tuebingen, Germany

**Keywords:** collective action, climate justice, interview, content analysis, self-efficacy

## Abstract

Student initiatives for climate justice are driving forces in the climate change debate, but the psychological determinants of students’ engagement for climate justice have hardly been investigated so far. For this study, we posited student engagement for climate justice to be a form of collective action and analyzed psychological determinants of collective action as well as subjective processes of change in these determinants. For this purpose, semi-structured interviews were conducted with four individuals who were engaged in different student initiatives. The results of a qualitative content analysis showed that student collective action for climate justice as reported by the respondents reinforced some of the psychological conditions of collective action established in the literature, such as collective and participatory self-efficacy expectations and feelings of fear and anger. We also found, however, that (first-time) participation in collective action cannot be fully explained by those known predictors. A sense of responsibility, awareness of problems, and extrinsic motives, such as social contact, were also conducive to participation, whereas politicized collective identities did not play a significant role. Finally, we discuss the results against the background of existing theoretical considerations and outline implications for further psychological study of collective action.

## Introduction

The analysis of psychological determinants for participation in collective action to bring about structural and sociopolitical changes is a central research area in environmental psychology research. This is especially true in light of the assumption that climate change, as a collective problem, also requires collective agency (van Zomeren et al., 2010; Rees and Bamberg, 2014). Student climate justice initiatives fit the psychological concept of collective action. Wright et al. (1990) define as collective action those actions in which people, as representative group members, advocate to improve the overall situation of their own group (see also Iyer and Leach, 2009). Collective action can also be performed by the privileged in solidarity with the disadvantaged (van Zomeren et al., 2011).

Drawing on the social identity model of collective action (SIMCA) by van Zomeren et al. (2008), we describe several extensions developed in the context of privilege engagement, or specifically, engagement for the climate justice movement. The SIMCA model represents an integrative approach that attempts to link three theoretical perspectives in the psychology of collective action: Instrumental theories (Olson, 1968; McCarthy and Zald, 1977; van Zomeren et al., 2010; Bamberg et al., 2015) are integrated together with emotion-based approaches (Smith and Ortiz, 2002; Iyer and Leach, 2009) and the social identity approach (Simon and Klandermans, 2001; Drury and Reicher, 2009) with the aim of describing predictors for participation in collective action. This means that this approach takes into account the extent to which particular actions are instrumental for the persons involved, that is, are means to achieve certain ends. At the same time, it takes into account that certain feelings and sensations guide the actions of the persons involved. Finally, a particular social identity in the sense of belonging to a certain social group plays a role in this context.

Accordingly, the model consists of the following three factors that interact to predict participation in collective action: (1) group-based outrage or anger as a result of a cognitive sense of injustice, (2) collective self-efficacy expectations as a belief that the group’s actions are meaningful and effective in relation to the intended goal, and (3) a social identity or politicized collective identity as identification with a social-political movement.

In this context, group-based emotions represent social emotions that are felt because of social identities. They form a conceptual bridge between group-based evaluations and action tendencies. According to van Zomeren et al. (2008), they serve to guide perceptions of injustice and motivate collective action. Politicized collective identity has both a direct and an indirect effect on the intention to participate in collective action *via* group-based outrage and collective self-efficacy expectations, because these can only arise through strong identification with the politically active group (Drury and Reicher, 2009).

The SIMCA model has been replicated in various sociopolitical contexts and adapted to the field of the climate justice movement (Rees and Bamberg, 2014; Barth et al., 2015), as climate justice activists from the Global North in most cases do not engage as a result of personally experienced grievances or disadvantages. They rather act as privileged individuals in solidarity with those who are first and most affected by the climate crisis. Barth et al. (2015) extend the SIMCA model to include the predictors of violated moral beliefs and a global identity. Moral beliefs refer to moral issues that are perceived as universal, objective, and unchanging (van Zomeren et al., 2011). Their violation motivates the attempt to restore conditions corresponding to those moral beliefs (Zaal et al., 2011) and enables the emergence of opinion-based groups. This means that shared opinion or at least belief in shared opinion about violated moral value standards is considered a criterion for collective action, resulting in the formation of an opinion-based group (Bliuc et al., 2007). Global identity means identification with all people of the globe regardless of nationality, appearance, or other criteria, in the sense of “identification with all humanity” (McFarland et al., 2013), which is considered in the literature as a direct precursor of global solidarity (Subašić et al., 2008).

The study presented here qualitatively investigated the psychological conditions of collective action. We also analyzed which subjective processes of change in psychological determinants of collective action emerged in the context of student engagement for climate justice. By this, we mean that we analyzed the extent to which respondents themselves were aware that the motivations for their engagement may have changed over time and the extent to which they reflected these processes of change. In doing so, the study examined to what extent existing theories on conditions of participation in collective action can be applied to the field of student engagement for climate justice and in which respect theoretical extensions seem to be necessary.

## Materials and Methods

In order to get a sense of the specific motivations that individuals engaged in climate justice would cite for their engagement, we chose to take a very open-ended research approach. The aim was to find out what committed activists name as motives for their respective actions and how they evaluate and reflect on their activities. Therefore, we applied a qualitative research design, which was characterized by a high degree of openness and reflexivity. Semi-structured interviews were conducted with student activists for climate justice. This form of interview is suitable for problem-related understanding of meaning. On the one hand, with the help of narrative-generating, guideline-supported questions, these interviews made it possible to reconstruct psychological processes of change of committed persons on the basis of subjective narratives (Whiting, 2008). On the other hand, by means of flexible and spontaneous follow-up questions, this method could also elaborate on less easily accessible subjective experiences or patterns of interpretation, the meaning of which can only be worked out through revealing dialogical work (Adams, 2015).

### Participants

Our aim was to integrate different and yet also typical cases into a sample. We formed counter horizons in the sample based on the type of initiative work; we distinguished between educational work and political work for climate justice, a distinction that is in line with the difference postulated by Rees and Bamberg (2014) between the intention to bring about individual behavioral change and the goal to bring about structural, sociopolitical, or institutional change. Using this criterion, we then selected several students actively engaged in climate justice work who fell into either both types of initiative work or only one of the two. Four people from different climate justice initiatives were interviewed in December 2020 (all personal data were pseudonymized): L. was a Master’s student in the first semester of the Global Change Management program; A. was a Master’s student in the first semester of the Agribusiness program; E. was studying English and politics for a teaching degree; and D. was studying law in the third semester.

### Data Collection and Analysis

The interviews were conducted by the first author of this article. The following basic principles were to be observed when conducting the interviews: As little guidance as possible, because the goal of the interviews was to get the interviewees to narrate freely; situational adjustment of the question sequence and wording; asking clarifying follow-up questions if necessary; no evaluative comments as interviewer; and only minimal expressions of empathy (such as nodding the head). The interview itself consisted of eight parts, each of which began with an introductory question: (1) “How did you get into your (first) initiative?” (2) “Tell me about the first day, your first time in your (first) initiative?” (3) “How is it today? Tell me about yourself and your initiative work today” (4) “How would you describe your progress in your initiative?” (5) “Looking back, what do you take away from your time in your initiative until today?” (6) “What will happen to you and your initiative in the future?” (7) “What do you have to say about climate change?” and (8) “What has not yet been asked and said that would be relevant today?” The interview guide also contained suggestions for possible follow-up questions that could be adapted to the situation. Taken as a whole, the questions aimed not only to capture the status quo but also to reflect subjective perceptions of developments.

For preparing the data analysis, the interviews were transcribed first. All names, places, initiative names, and other personal details were pseudonymized. Then, a qualitative content analysis was conducted. The first analysis step consisted mainly of hermeneutic-interpretive text work, with the goal of a general basic understanding. Summaries were written in the margins for individual sections of meaning. Initial thoughts and ideas about hypotheses and theoretical connections were recorded in memos. The conclusion of the initiating text work was the writing of short descriptive case summaries, which, in addition to a kind of one-sentence description, also contained keyword-like summaries. Subsequently, main categories were formed, which were composed inductively from the initiating text work as well as from the deductively obtained categories (e.g., politicized collective identity or collective self-efficacy expectations) already established in advance. In the coding guide, the categories were supplemented with short definitions and prototypical examples. This was followed by a coding process, in which text sections were assigned to matching categories. The same text section could be coded several times. In a further step, the aim was to form inductive subcategories, to create new main categories, and, if necessary, to subsume other codes from the first coding process, as few simple categories as possible were to be formed, which were nevertheless as differentiated as necessary to answer the research questions. Finally, we conducted a second coding run with all of the categories, resulting in an aggregation of categories.

## Results

In the following sections, we first present the psychological categories of student engagement that emerged from the interviews. In line with Stürmer and Simon (2004), we distinguished between internal and external categories.

### Internal Psychological Categories of Student Engagement

*Violated moral convictions* were only addressed once across all interviews: In the context of the commitment to veganism, participant E. did not explicitly formulate a basic moral stance, but implied that ethical values were violated by animal husbandry and interpreted this as a condition of participation in collective action.

From the theoretical perspective outlined above, *social identities* can be addressed in the context of politicized social identities or as global identification linked to an attitude of solidarity toward those suffering from the climate crisis. A *politicized social identity* could only be coded specifically in relation with E.’s first-time participation in collective action. At the time of the interviews, however, all of the interviewees spoke of their own initiatives in the “we” form, so that at some points it was necessary to ask more detailed questions whether the interviewees were talking about activities in which they themselves were involved or which were supported by other group members. Apparently, they had strongly internalized their group membership and acted as representatives of their initiatives. Based on their involvement, they had developed a kind of ownership of their projects (Greving et al., 2020). An attitude of solidarity toward people suffering from climate change, as discussed in the literature, could not be coded in any of the interview transcripts.

*Self-efficacy expectations* were formulated in the interviews as collective, participatory, and limited self-efficacy expectations as well as in the category of self-confidence. *Collective self-efficacy expectations*, in the sense of the assumption that the collective action of an initiative can achieve something to address the challenges of the climate crisis, were named in particular in the context of the start of engagement. Furthermore, the interviews provided a lot of text passages expressing a subjective change of collective self-efficacy expectations. During the evaluation process, a differentiation was made between self-efficacy experiences and expectations. For example, D. reported the realization of being able to achieve more than one might think after all. *Participatory self-efficacy expectations* could not be coded in relation to respondents’ first-time participation in collective action. However, respondents expressed that they felt over time that their own input was indispensable. In addition, respondents also had future-related expectations that they could accomplish things themselves and that “maybe even something will change” as a result. In this context, some of the expectations formulated related less to one’s own contribution to collective action and more to the effectiveness of individual projects and ideas. This illustrates that respondents were clear about which actions, projects, and strategies were instrumental in achieving their goals. In addition to experiencing their own effectiveness, the interviewees also reported *limited self-efficacy expectations*, in the sense of the limits to their own possibilities for action and impact. E., for example, did not believe that people would change their climate-damaging behavior and viewed initiatives of the climate justice movement as having too little impact. This was accompanied by expressions of a sense of hopelessness. Thus, on the one hand, respondents experienced that their own actions within the initiative could be successful, but they also expected that the successes could only produce small changes in the overall global context against the backdrop of the complexity of the climate crisis. Another category that could be inductively extracted from the data material during the coding processes was *self-confidence*. At various points in the interviews, L., A., and D. noted that they had become more confident in the course of the initiative. For example, they had been able to have discussions with important stakeholders at the universities, they had gained confidence in putting forward their own opinions at student parliament meetings, and they had learned to admit to making mistakes.

*Group-based emotions* were also addressed, distinguishing between anger, fear, and hope. Participants reported the motivating nature of *anger* at the great injustice of the climate crisis. However, respondents’ anger also changed over time. Anger, which played a major role at the beginning of their engagement, developed and became less (E.) or even more relevant (L. and A.). *Fear* also seemed to undergo a subjective change over the course of the engagement. Collective fear of the consequences and inevitability of climate change increased the more deeply respondents engaged with the issue. In this context, the participants referred to moments of despair. In addition, some passages expressed a collective sense of stress that seemed to be related to the experience of limited effectiveness and a collective fear of an uncertain future. There was also a change in the pleasant group-related emotion of *hope*. Participants reported more hope in connection with the experience of working together with like-minded people who also had the will to make a difference. This was not necessarily accompanied by the hope of ultimately being able to really achieve their climate policy goals. In connection with the initial phases of the interviewees’ involvement, no other emotions besides those mentioned, such as feelings of guilt, came up.

Another category that could be extracted inductively from the material was the code *sense of responsibility*. A sense of responsibility already played a role at the beginning of the engagement but tended to become stronger over time. Participants felt that the urgency of the problem was growing, and their involvement was becoming correspondingly more important. The interviewees also had an extraordinary *awareness of the problem*; they were highly aware of the anthropogenic causes, dynamics, and likely consequences of climate change. They were able to provide very detailed and differentiated information on the subject.

The interviewees also gained *practice-related transfer knowledge* over the course of the initiative period that could be applied in other contexts or other forms of collective action. They gained experience in working in a team, in project and event management, and in rhetorical expression. In addition, change was expressed in another subcategory, *mindfulness*, which was also assigned to the main category of transfer knowledge, because of its transferability to other areas of life. Three of the interviewees reported phases in their engagement in which they had clearly exceeded their own stress limits and had overworked themselves. As a result, the initiatives had started to regularly check the capacities of the members and were currently very mindful of personal resources.

### External Psychological Categories of Student Engagement

In the interviews of D. and A, it became clear that an *invitation* by friends or roommates to participate in collective action played a central role in the beginning of their engagement for climate justice. L. and E., on the other hand, began their engagement on their own initiative. Three of the four respondents indicated that, starting from their first initiative, they moved from one project to the next, from one collective action to another. This indicated intensive networking among the various initiatives fighting for climate justice and an increasing degree of personal networking with other activists, through which many opportunities for engagement became visible. Subjectively, the respondents perceived it as a *coincidence* that they had become active in precisely these initiatives.

Another motive for their involvement, which was regularly mentioned in the interviews, was the desire to *meet new people* and the opportunity to exchange ideas with others. L. argued in the interview that universities should make even more use of the fact that students are ready to “leap into life” and that students could be easily motivated to become active. A. also emphasized the potential of attracting young people to the topic of climate justice at universities. Furthermore, they referred to their own initial *openness* to initiatives, projects, and ideas as a subjectively perceived condition for the beginning of their involvement and also for having gradually joined new projects.

The interviewees portrayed the initiatives as places where “you are more likely to be forgiven for mistakes.” The respondents could try themselves out in this kind of *experimental space* and devote themselves time and again to new fields of activity. There also seemed to be a great deal of mutual trust in many initiatives; newcomers were directly involved in the initiative’s efforts, work was done on an equal footing, and decisions were made by consensus. The interviewees emphasized that they had met nice, open people in the initiatives, “with whom one could feel comfortable very quickly.” They liked being surrounded by *like-minded people* who shared similar views, with whom they could exchange ideas and by whom they felt inspired. Friendships formed were even mentioned as a particularly important aspect in terms of what participants would take away from their involvement.

## Discussion

In the following, we discuss in which of the categories identified here the theoretical considerations on participation in collective action presented above were reflected and to what extent our findings could extend the theories. Moreover, the subjective processes of change in the psychological categories throughout the interviewees’ engagement period are considered from a theoretical point of view.

It is striking that violated moral beliefs did not seem to play a major role for the respondents. What did emerge, however, when looking back at the beginning of their engagement, was the respondents’ great awareness of problems at that time, which apparently constitutes a condition for people to engage in student collective action for climate justice. In this context, awareness of the problem could possibly precede personal moral convictions. When people inform themselves about the causes and consequences of the climate crisis, they may recognize along the way that their basic moral values (e.g., the value of justice) were being violated. This could be the reason why problem awareness does not play a role in current models of collective action, because awareness is already implicitly included as a prerequisite of violated moral beliefs. Future research could benefit from taking a closer look at this distinction. It is also notable that an attitude of solidarity toward those suffering from climate change as a result of a global identity (Barth et al., 2015) could not be coded in any of the transcripts. However, this may also be a result of the data collection method used, in which the questions were predominantly related to the specific engagement of the respondents, making identity as an activist more salient than global identity.

Collective self-efficacy expectations appear to be beneficial to students joining student initiatives for collective action. This instrumental aspect of our findings is consistent with the existing literature, in which the expectation that group action can do something to address grievances is one of the important and often replicated predictors of participation in collective action (Klandermans, 1997; van Zomeren et al., 2008, 2010; van Stekelenburg and Klandermans, 2013). The fact that no text passages could be coded in the category of participatory self-efficacy expectations suggests that this category might not be too relevant for newcomers to student initiatives. There are corresponding findings from studies by Bamberg et al. (2015) and Mazzoni et al. (2015), showing that participatory and collective self-efficacy expectations have different relevance for newcomers with the intention of participating, compared to those already involved. Such expectations are predictive of ongoing participation in collective action, especially for established activists.

The finding that being angry may have promoted engagement is also consistent with studies in collective action research, such as van Zomeren et al.’s (2008) SIMCA model and Barth et al.’s (2015) findings postulating the predictive value of group-based outrage for collective action participation (for a review, see van Zomeren, 2013). Thomas et al. (2009) also considered outrage directed at a system or at those responsible for the system to be particularly conducive for participation in social-political movements to overcome social injustices. Shared outrage toward third parties or the system causes group boundaries between the privileged and the disadvantaged to crumble and reinforces the norm of fighting together for structural change. The fact that no text passages with the deductive category of guilt could be coded at the respondents’ start of engagement also goes hand in hand with Thomas et al.’s (2009) postulate that guilt tends to motivate “fig leaf” actions, which was not the case for the respondents, since they were seriously and strongly committed to their engagement. It is possible, however, that collective guilt was also implicitly expressed in the sense of responsibility category. Collective fear, in contrast to outrage, did not seem to contribute to a commitment to solve the threat of climate change, which contradicted the existing literature about the effects on participation in collective action of experimentally manipulated fear (van Zomeren et al., 2010).

Based on the findings, it can be assumed that the sense of responsibility, which plays a central role in theories of individual environmental protection behavior, is also an important condition of participation in collective action for climate justice. It is noteworthy that this concept is not a stand-alone predictor in current models of participation in collective action, especially in those models that attempt to explain why privileged people advocate for structurally disadvantaged people in protests and initiatives. Barth et al. (2015), however, define the concept of solidarity of the privileged toward those suffering from the climate crisis as a form of a collective sense of responsibility. Thomas et al. (2009), on the other hand, argue that group-based feelings of guilt or moral outrage go hand in hand with the attribution of responsibility to one’s in-group and that once this attribution becomes a feature of collective identity, certain action-related norms become salient. Rees and Bamberg (2014), in turn, postulate that a collective sense of guilt leads to collective protest primarily when moral beliefs concerning the in-group are violated. It is possible that a sense of responsibility represents a condition of collective action that has always been considered implicitly by researchers. But it might be worth taking it into account as a stand-alone condition in order to position a sense of responsibility within the models and examine it in its interdependencies with the other predictors.

External factors were also found to be relevant in this study. In particular, the influence of both injunctive and descriptive social norms of participation emerged. The social environment greatly influenced which initiatives the respondents were involved in. The extended SIMCA model (van Zomeren et al., 2011) does not present social norms as a stand-alone predictor, but it is inherent in the predictor of (politicized) social identity in the model. Other researchers (Stürmer and Simon, 2004; Rees and Bamberg, 2014) postulated that social norms are a strong predictor of participation in collective action, or at least mediate the predictive influence of social identities on participation intention.

Finally, the findings suggest that the phases of self-discovery, upheaval, or reorientation (such as at the start of one’s studies), together with extrinsic motives, such as social contact, may be particularly conducive to the beginning to engage in collective action for climate justice. In this study, interestingly, three of the respondents started getting involved right at the beginning of their studies. This assumption is in line with environmental psychological findings on individual behavioral changes, which indicate that the life phases of upheaval are particularly suitable for breaking old habits and getting used to new ones (Bridges and Bridges, 2019).

This study has several limitations. First, its findings are based on a small and highly selective sample that, nevertheless, was reasonably characteristic of the population of students engaged in collective action for climate justice. In addition, the interaction between interviewer and interviewee was determined by social dynamics that may have influenced the responses. Thus, qualitative data from interviews were not immune to socially desired responses. Moreover, the qualitative content analysis can be viewed critically, as it may have overlooked important issues and may have come to inaccurate conclusions. Finally, the results of this work cannot be used to prove or disprove any cause-effect relationships empirically. The qualitative, partly retrospective survey method, which is not protected from socially desirable responses, cannot exclude the effect of confounding variables on the transformation of psychological conditions.

On the other hand, the study also has a number of relevant strengths. Our findings are able to integrate relevant qualitative and quantitative research. Our research shows which aspects of existing theories were also reflected in our data and in which respects existing approaches should possibly be extended. Our methodological approach has helped to identify individual and group-related motives, emotions, and intentions to act that would not have been taken into account in purely theory-based quantitative studies. The psychological determinants of engagement in collective action identified here can be taken up and empirically investigated in further qualitative and quantitative studies. This in turn can and should lead to meaningful modifications of relevant theories and models of collective action.

## Data Availability Statement

The datasets presented in this article are not readily available because based on the description of the participants and the projects in which they were involved, identification of the participants cannot be completely ruled out. For this reason, it is necessary to refrain from publishing the interview data. Requests to access the datasets should be directed to j.kimmerle@iwm-tuebingen.de.

## Ethics Statement

Ethical review and approval was not required for the study on human participants in accordance with the local legislation and institutional requirements. The participants provided their written informed consent to participate in this study.

## Author Contributions

HB and JK contributed to the conceptualization and design of the study, and the writing of the manuscript. HB conducted the interviews and the analyses. JK supervised and provided feedback, funding, and resources. All authors contributed to the article and approved the submitted version.

## Conflict of Interest

The authors declare that the research was conducted in the absence of any commercial or financial relationships that could be construed as a potential conflict of interest.

## Publisher’s Note

All claims expressed in this article are solely those of the authors and do not necessarily represent those of their affiliated organizations, or those of the publisher, the editors and the reviewers. Any product that may be evaluated in this article, or claim that may be made by its manufacturer, is not guaranteed or endorsed by the publisher.
